# Admission Cytokine Profiling for ICU Mortality Prediction in Heterogeneous Acute Respiratory Failure: An Exploratory Cytokine Profiling Study

**DOI:** 10.3390/diagnostics16121814

**Published:** 2026-06-12

**Authors:** Joonho Lee, Jae-Hoon Ko, Hyunseung Nam, Jaeyoung Choi, Jin Yang Baek, Miryeo Nam, Chi Ryang Chung, Jeong Hoon Yang, Gee Young Suh, Ryoung-Eun Ko

**Affiliations:** 1 Department of Critical Care Medicine, Samsung Medical Center, Sungkyunkwan University School of Medicine, Seoul 06351, Republic of Korea; 2jh00026@gmail.com (J.L.); jychoi1213@hanmail.net (J.C.); ccrzzang@gmail.com (C.R.C.); jhysmc@gmail.com (J.H.Y.); smccritcare@gmail.com (G.Y.S.); 2 Division of Infectious Disease, Department of Medicine, Samsung Medical Center, Sungkyunkwan University School of Medicine, Seoul 06351, Republic of Korea; jaehoon.ko@gmail.com (J.-H.K.); jy34.baek@gmail.com (J.Y.B.); 3 Department of Critical Care Medicine, Hallym University Sacred Heart Hospital, Anyang 14068, Republic of Korea; namhs2001@naver.com; 4 Asia Pacific Foundation for Infectious Diseases (APFID), Seoul 06367, Republic of Korea; 5 Department of Clinical Research Design and Evaluation, Samsung Advanced Institute for Health Sciences & Technology (SAIHST), Sungkyunkwan University School of Medicine, Seoul 06351, Republic of Korea; nmr1010@hanmail.net; 6 Division of Cardiology, Department of Medicine, Samsung Medical Center, Heart Vascular Stroke Institute, Sungkyunkwan University School of Medicine, Seoul 06351, Republic of Korea

**Keywords:** acute respiratory failure, cytokines, biomarkers, prognosis, intensive care unit

## Abstract

**Background/Objectives**: Acute respiratory failure (ARF) encompasses heterogeneous etiologies, and early bedside prognostication remains challenging. Cytokines and chemokines may capture underlying biological severity and identify high-risk patients. We evaluated whether admission cytokine/chemokine profiles add incremental prognostic value over clinical risk factors in unselected ARF patients. **Methods**: This prospective, single-center cohort study enrolled adult patients admitted to medical ICUs with ARF requiring high-intensity respiratory support. Plasma samples were collected within 24 h of ARF diagnosis, and 19 cytokines/chemokines were measured using multiplex immunoassays. The primary outcome was ICU mortality. Univariate and multivariable logistic regression models assessed associations between biomarkers and mortality, with discrimination evaluated by the area under the receiver operating characteristic curve (AUC). **Results**: Among 41 patients, 15 (37%) died in the ICU. Non-survivors had higher rates of immunosuppression (80% vs. 38%, *p* = 0.010) and hematologic malignancy (67% vs. 31%, *p* = 0.026). CXCL10 (IP-10), IL-18, and CCL2 (MCP-1) were significantly higher in non-survivors, and IL-1Ra showed a marked numerical increase with a significant univariable association with ICU mortality, despite comparable severity scores and oxygenation indices at admission. A clinical core model (SOFA, immunosuppression, hematologic malignancy) achieved an AUC of 0.74 (95% CI 0.58–0.90); adding cytokines improved discrimination modestly (AUC 0.76–0.80). In highest-quartile survival analyses, IL-1Ra (*p* = 0.002), CXCL10 (*p* = 0.005), and CCL2 (*p* = 0.009) demonstrated significant survival separation. **Conclusions**: At ICU admission, CXCL10 (IP-10), IL-18, CCL2 (MCP-1), and IL-1Ra showed exploratory associations with ICU mortality and were prioritized as candidate inflammatory biomarkers. These findings require validation in larger multicenter cohorts.

## 1. Introduction

Acute respiratory failure (ARF) is a common reason for intensive care unit (ICU) admission and initiation of high-intensity respiratory support. In real-world practice, clinicians often must stabilize oxygenation and ventilation before the exact etiology is fully clarified, because ARF can arise from heterogeneous processes [1]. Acute respiratory distress syndrome (ARDS) definitions such as the Berlin definition provide an important framework for research standardization [2]. However, a substantial proportion of ICU ARF patients do not fit neatly into a single category at presentation, and early bedside prognostication remains challenging across this broader ARF spectrum.

Blood-based biomarkers have been explored as tools to complement clinical variables by capturing biological severity not apparent from clinical variables alone. A systematic review and meta-analysis of plasma biomarkers in ARDS highlighted that multiple circulating markers show associations with outcomes, suggesting that biologically informed risk stratification may be feasible [3]. Among these, markers reflecting endothelial activation (e.g., angiopoietin-2) and alveolar epithelial injury (e.g., soluble RAGE) have demonstrated reproducible associations with disease severity and mortality across studies and meta-analyses [4,5]. These observations motivate the evaluation of whether early biomarker profiles can improve risk assessment beyond routine clinical scoring, particularly in mixed, diagnostically uncertain ARF populations where decisions about monitoring intensity, escalation, and goals-of-care discussions must often be made early.

Within inflammatory biomarkers, cytokines and chemokines are especially attractive candidates because they can reflect early host-response intensity and may identify patients at heightened risk. In large ARDS trial cohorts, plasma interleukin (IL)-18 provided incremental prognostic information and identified a subset of patients with high mortality risk even when traditional inflammatory marker patterns would otherwise classify them as lower-risk [6,7,8]. Consistently, in a sepsis-induced ARDS statin trial, higher IL-18 and rising IL-18 over time were associated with increased mortality [7]. Chemokine signaling has also shown prognostic relevance in severe viral respiratory illness: early plasma CXC chemokine ligand [CXCL10 (interferon-inducible protein (IP)-10)] and related chemokines predicted ICU admission or death in severe acute respiratory syndrome [9], and in coronavirus disease 2019 (COVID-19), plasma IP-10 has been strongly associated with severity and progression [10]. However, the prognostic utility of cytokine/chemokine markers in unselected, heterogeneous ARF populations remains unclear. We therefore conducted an exploratory analysis measuring a broad panel of cytokines/chemokines at ICU admission to identify candidate biomarkers with incremental value over clinical risk factors for future validation.

## 2. Materials and Methods

### 2.1. Study Design and Patient Selection

**Figure 1:**
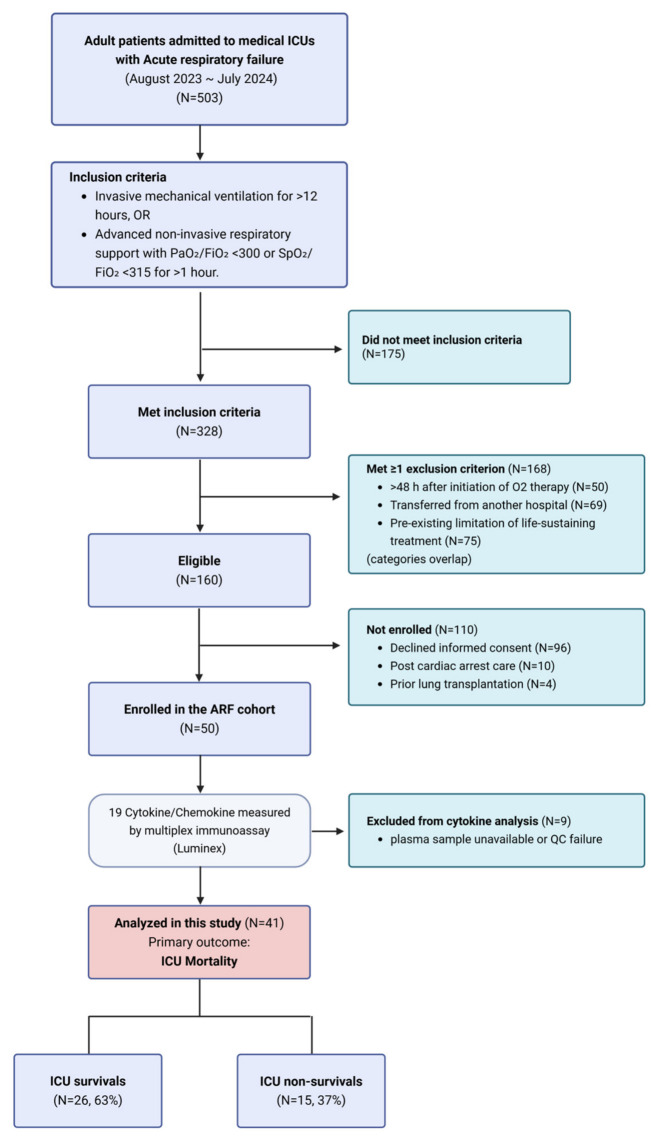
Study flow diagram. Adult patients admitted to medical ICUs with ARF between August 2023 and July 2024 were screened. Inclusion criteria included invasive mechanical ventilation for more than 12 h, or advanced non-invasive respiratory support with PaO_2_/FiO_2_ ratio below 300 or oxygen saturation by pulse oximetry to FiO_2_ ratio below 315 for more than 1 h. Fifty patients were enrolled, of whom 41 had plasma samples available and were included in the present cytokine analysis (26 [63%] survived to ICU discharge and 15 [37%] died in the ICU). Plasma samples were analyzed using multiplex immunoassay (Luminex) to measure 19 cytokines/chemokines. ARF, acute respiratory failure; ICU, intensive care unit.

We conducted a single-center prospective cohort study and consecutively enrolled adult patients admitted to the medical ICUs with ARF. The study was registered at ClinicalTrials.gov (NCT06479421, registered 18 August 2023). Patients were eligible if they received invasive mechanical ventilation through an endotracheal tube or tracheostomy for more than 12 h, regardless of whether they fulfilled specific ARF criteria, as prolonged invasive mechanical ventilation itself indicates clinically significant respiratory failure. In addition, patients requiring advanced respiratory support without invasive mechanical ventilation, including high-flow nasal cannula or noninvasive ventilation using bilevel positive airway pressure or continuous positive airway pressure with an oronasal or facial mask, were included if they demonstrated ARF characterized by either a partial pressure of arterial oxygen (PaO_2_)/fraction of inspired oxygen (FiO_2_) ratio below 300 or an oxygen saturation by pulse oximetry to FiO_2_ ratio below 315 for more than 1 h [11,12]. Patients on chronic home oxygen or chronic ventilatory support without an acute escalation for the index ICU admission were not enrolled. Blood samples were obtained at study enrollment (within 24 h of ARF diagnosis) for cytokine measurement. Clinical data including demographics, comorbidities, illness severity indices, and outcomes were collected from the medical record. The primary outcome was ICU mortality, defined as death before ICU discharge. The study protocol was approved by the institutional review board, and informed consent was obtained from patients or surrogates as required (IRB no. 2023-08-003-009). The study flow is presented in Figure 1.

### 2.2. Cytokine Measurements

Blood samples were collected into ethylenediaminetetraacetic acid anticoagulant tubes and centrifuged at 1200× *g* for 15 min at room temperature to obtain plasma. Plasma samples were stored at −80 degrees C until analysis and subjected to no more than two freeze–thaw cycles. To minimize batch or plate effects, samples were assayed on the same plate whenever possible.

We quantified 19 cytokines and related biomarkers using magnetic bead-based multiplex assays on the Luminex platform (R&D Systems, Minneapolis, MN, USA) as follows: (1) a 13-plex cytokine panel (Performance Human XL Cytokine Panel) including CCL2, CCL3, CCL4, CCL5, CXCL10, interferon-gamma, IL-1Ra, IL-2, IL-4, IL-6, IL-13, IL-17A, and tumor necrosis factor (TNF)-alpha; (2) a 5-plex inflammatory panel (Human Discovery Assay) including angiopoietin-2, IL-10, IL-18, IL-23, and soluble RAGE; and (3) a single-plex assay for cancer antigen 15-3 (CA15-3). Samples were diluted per manufacturer instructions (1:2 for 13-plex and 5-plex; 1:50 for CA15-3). Lot-specific lower limit of quantification (LLOQ) and upper limit of quantification were obtained from Certificates of Analysis and adjusted for dilution. Analyte concentrations are reported in pg/mL. Values below the LLOQ were imputed as LLOQ/2 to allow log2 transformation. Analytes with more than 30% of values below the LLOQ were excluded from the primary regression analyses [13].

### 2.3. Statistical Analysis

This was an exploratory study; sample size was determined by feasibility rather than formal power calculations. Cytokine/chemokine concentrations were log2-transformed, and effect estimates reflect risk change per doubling of concentration. Baseline characteristics were compared using the Wilcoxon rank-sum test for continuous variables and chi-squared or Fisher’s exact test for categorical variables.

For each biomarker, we fitted a univariate logistic regression model and reported the odds ratio (OR) with its 95% confidence interval and the area under the curve (AUC). Multiplicity was addressed using the Benjamini–Hochberg false discovery rate adjustment. Bonferroni correction was additionally applied as a conservative sensitivity analysis. Four biomarkers (CXCL10, CCL2, IL-18, and IL-1Ra) meeting the LLOQ threshold and *p* < 0.05 in unadjusted analysis were selected for multivariable evaluation, with the false discovery rate-adjusted q values also reported. We built a core model that included the Sequential Organ Failure Assessment (SOFA) score, immunosuppression, and hematologic malignancy, and added each cytokine one at a time to respect the limited events-per-variable ratio. The optimism-corrected area under the curve (AUC), net reclassification improvement, and integrated discrimination improvement were estimated using bootstrap resampling, with 95% confidence intervals. Time to ICU death was analyzed using Kaplan–Meier curves with censoring at ICU discharge. Patients were stratified by a pre-specified cytokine quartile (Q4 vs. Q1–Q3 combined) and compared using the log-rank test. As a complementary analysis, Cox proportional-hazards regression was performed using each cytokine as a continuous variable (per log2 doubling). The association between PaO_2_/FiO_2_ ratio and cytokine concentrations was assessed using Spearman correlation. All tests were two-sided. Exact *p* values are reported, with statistical significance set at *p* < 0.05. Analyses were performed using R software (version 4.4.2, R Foundation for Statistical Computing, Vienna, Austria).

## 3. Results

### 3.1. Patient Characteristics

During the enrollment period (August 2023 to July 2024), 503 patients admitted to the medical ICU were prospectively logged for ARF screening. Of these, 328 met the inclusion criteria and 160 remained eligible after applying exclusion criteria. Fifty patients consented and were enrolled, of whom 41 had plasma samples available for the present cytokine analysis (Figure 1). Of these 41 analyzed patients, 26 (63%) survived to ICU discharge and 15 (37%) died in the ICU. Median age was similar between groups (65 [55–71] vs. 66 [61–71] years, *p* = 0.800), and illness severity tended to be higher among ICU non-survivors (Simplified Acute Physiology Score-3 60 [48–75] vs. 68 [62–77], *p* = 0.078), while SOFA scores did not differ significantly (10.0 [7.0–11.0] vs. 11.0 [7.0–16.0], *p* = 0.300). Immunosuppression and hematologic malignancy were more frequent among ICU non-survivors (80% vs. 38%, *p* = 0.010; 67% vs. 31%, *p* = 0.026, respectively). Oxygenation at admission was comparable (P/F ratio 178 [123–226] vs. 218 [114–370], *p* = 0.400), and P/F ratio category distributions did not differ significantly (*p* = 0.200). Hospital length of stay was shorter in ICU non-survivors (16 [6–28] vs. 25 [17–39] days, *p* = 0.032) (Table 1). The median interval from ICU admission to plasma sampling was 17.8 h (interquartile range 6.8 to 21.1). Baseline characteristics by day 0 respiratory support modality are presented in Supplementary Table S1.

### 3.2. Cytokine/Chemokine Profiles

Several inflammatory mediators measured at ICU admission were higher in ICU non-survivors (Table 2). While non-survivors showed a trend toward higher levels across most cytokines and chemokines, significant group differences were observed primarily among monocyte/macrophage-derived mediators (CCL2, CXCL10, IL-18), whereas IL-1Ra showed a marked numerical increase and was significantly associated with mortality in univariable logistic regression. T cell-associated cytokines (IFN-γ, IL-2, IL-4, IL-17A) did not reach statistical significance despite numerical differences.

In exploratory subgroup analyses, effect directions were generally similar across infectious versus non-infectious and immunocompromised versus non-immunocompromised. No cytokine-by-subgroup interaction reached statistical significance; however, the estimates were imprecise because of the small number of events, particularly in the non-immunocompromised subgroup (Supplementary Table S2).

### 3.3. Univariate Screening and Multivariable Prognostic Models

In univariate logistic regression, clinical risk factors associated with ICU mortality included immunosuppression (OR 6.40 [1.58–33.56], *p* = 0.015) and hematologic malignancy (OR 4.50 [1.21–18.86], *p* = 0.030) (Table 3). Among biomarkers, higher CXCL10 (IP-10) (OR 1.54 [1.12–2.27], *p* = 0.015), IL-18 (OR 1.61 [1.08–2.57], *p* = 0.027), CCL2 (MCP-1) (OR 1.53 [1.08–2.25], *p* = 0.022), and IL-1Ra (OR 1.43 [1.03–2.05], *p* = 0.040) showed the strongest univariable associations with ICU mortality and were prioritized for multivariable model augmentation (Table 3; Supplementary Table S3).

For discrimination, a SOFA-only model showed modest performance (AUC 0.60 [0.41–0.79]). The Clinical Core model (SOFA + immunosuppression + hematologic malignancy) improved discrimination (AUC 0.74 [0.58–0.90]) (Table 4). Adding individual biomarkers further improved discrimination, with AUCs of 0.79 for CXCL10, 0.76 for IL-18, 0.80 for CCL2, and 0.78 for IL-1Ra. Calibration was acceptable across models (Hosmer–Lemeshow *p* > 0.2). In the univariable logistic screening, no cytokine remained significant after BH correction (smallest q = 0.082; Supplementary Table S4).

Internal validation using 1000 bootstrap iterations with Harrell’s optimism correction yielded an optimism-corrected AUC of 0.68 for the core model and 0.67–0.71 for the cytokine-augmented models (Supplementary Table S5). The continuous net reclassification improvement ranged from 0.39 to 0.66 and the integrated discrimination improvement from 0.03 to 0.07, with all net reclassification improvement confidence intervals crossing zero.

A sensitivity analysis excluding patients with hematologic malignancy preserved the direction of cytokine–mortality associations, although no cytokine reached nominal significance in this subset (Supplementary Table S6).

### 3.4. Time-to-ICU-Death Analyses

**Figure 2:**
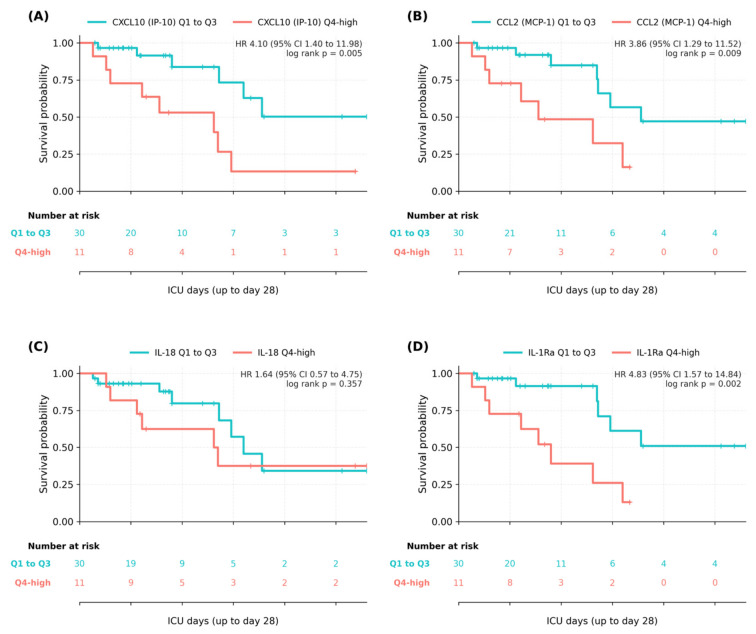
Kaplan–Meier survival curves for ICU mortality stratified by quartiles of candidate cytokines. Patients were stratified into quartiles (Q1–Q4) based on admission cytokine concentrations. Survival curves compare the highest quartile (Q4, red) versus the lower three quartiles combined (Q1–Q3, blue). Time zero represents ICU admission; patients were censored at ICU discharge. (**A**) CXCL10 (IP-10): Q4 vs. Q1–Q3, log-rank *p* = 0.005. (**B**) CCL2 (MCP-1): Q4 vs. Q1–Q3, log-rank *p* = 0.009. (**C**) IL-18: Q4 vs. Q1–Q3, log-rank *p* = 0.357. (**D**) IL-1Ra: Q4 vs. Q1–Q3, log-rank *p* = 0.002. IL-1Ra, CXCL10, and CCL2 demonstrated significant survival separation concentrated in the highest concentration quartile. Abbreviations: ICU, intensive care unit; CXCL10, C-X-C motif chemokine ligand 10 (IP-10); CCL2, C-C motif chemokine ligand 2 (MCP-1); IL-18, interleukin-18; IL-1Ra, interleukin-1 receptor antagonist. Tick marks on the survival curves indicate censored observations.

In Kaplan–Meier analyses comparing Q4 versus Q1–Q3 combined, three of the four candidate biomarkers demonstrated clear survival separation within 28 days. IL-1Ra showed the most pronounced separation (log-rank *p* = 0.002), followed by CXCL10 (IP-10) (*p* = 0.005) and CCL2 (MCP-1) (*p* = 0.009). In contrast, quartile-based survival differences were not significant for IL-18 (*p* = 0.357) (Figure 2). These findings suggest that mortality risk is concentrated in the highest concentration range for select inflammatory mediators, with IL-1Ra and chemokines CXCL10 and CCL2 demonstrating the most robust threshold-based risk stratification patterns.

As a complementary analysis, we performed Cox proportional-hazards regression using each cytokine as a continuous variable. No analyte remained significant after BH correction among those that passed the LLOQ filter (smallest q = 0.066 for IL-1Ra, CCL2, and soluble RAGE; Supplementary Table S7).

### 3.5. Relationship Between Oxygenation Impairment and Inflammation

PaO_2_/FiO_2_ (P/F) ratio at ICU admission showed only weak monotonic associations with the prognostic biomarkers. Spearman’s rho was 0.15 for CXCL10 (*p* = 0.340) and 0.21 for IL-18 (*p* = 0.190), with similarly small effect sizes for CCL2 and IL-1Ra. When stratified by conventional P/F categories (≤100, 101–200, 201–300, >300), biomarker distributions showed substantial overlap with wide within-category dispersion and no consistent graded trend (Supplementary Figure S1). Collectively, these findings suggest that the cytokine signal is not simply a surrogate for concurrent oxygenation impairment.

## 4. Discussion

In this exploratory study of ICU patients with ARF, we evaluated whether admission cytokine/chemokine profiles could identify patients at higher risk of ICU mortality and add incremental prognostic value beyond a simple clinical model. Several mediators were elevated in non-survivors, with CXCL10 (IP-10), CCL2 (MCP-1), IL-18, and IL-1Ra emerging as candidate prognostic markers. When added to a core model that included SOFA score, immunosuppression, and hematologic malignancy, these cytokines produced modest AUC improvements while calibration remained acceptable.

These findings have potential clinical relevance for early ARF management. Clinicians often must stabilize patients before etiologies are clarified, and biomarkers supporting rapid risk stratification could help prioritize monitoring intensity and inform goals-of-care discussions [14]. This need may be particularly acute in immunocompromised patients, who comprised 80% of non-survivors and represent a growing high-risk ICU population [15,16,17]. Effect directions of these cytokines were preserved within the immunocompromised subgroup, although point estimates were imprecise (Supplementary Table S2), so the prognostic utility within this high-risk subgroup requires confirmation in larger studies.

A key observation is that significant cytokine elevations were confined to monocyte/macrophage-derived mediators (CCL2, CXCL10, IL-1Ra, IL-18), while T cell-associated cytokines did not differ between survivors and non-survivors. The observable signal was concentrated in monocyte/macrophage-derived mediators, consistent with the early phase of critical illness when innate immunity predominates [18,19] and emergency myelopoiesis is engaged [20,21,22]. Notably, IL-2, IL-4, and IL-17A had more than 85% of values below the LLOQ, so our data cannot definitively address T-cell-mediated contributions, and the apparent myeloid-biased pattern should be interpreted with this assay limitation in mind. Beyond these assay-related considerations, the preserved direction of association after excluding patients with hematologic malignancy suggests that the observed signal was not explained solely by hematologic malignancy, although residual confounding remains possible.

Among individual biomarkers, IL-1Ra showed the strongest survival separation. Elevated IL-1Ra paradoxically indicates intense inflammatory activation, as its production is proportional to upstream IL-1 signaling; high IL-1Ra has been associated with increased mortality in sepsis and ARDS [23,24]. CXCL10 has consistently predicted adverse outcomes in severe viral pneumonia, including severe acute respiratory syndrome and COVID-19 [9,10]. Our finding of a high-tail survival separation pattern (Q4 vs. Q1–Q3) suggests a subset of ARF patients may enter a high-risk inflammatory trajectory detectable at admission. CCL2, a key monocyte chemoattractant implicated in ARDS pathogenesis, also demonstrated significant survival separation [10]. IL-18 showed strong univariate signal, though quartile-based survival comparison did not reach significance (*p* = 0.357) [6,7], possibly reflecting limited power. Cytokine panel studies in COVID-19-associated ARDS support multi-marker rather than single-marker approaches [25,26,27].

An additional finding underscores the complementary role of inflammatory biomarkers. At ICU admission, survivors and non-survivors did not differ significantly in clinical severity scores (SAPS-3, SOFA) or oxygenation impairment. Biomarkers of endothelial activation (angiopoietin-2) and alveolar epithelial injury (soluble RAGE, CA15-3) [3,4,5,28] showed numerically higher values in non-survivors but did not reach statistical significance. In contrast, monocyte/macrophage-derived mediators were significantly elevated, suggesting that early innate immune activation may more sensitively identify high-risk patients than traditional severity assessments [29]. Two patients with similar hypoxemia can exhibit markedly different inflammatory profiles, underscoring the potential value of integrating inflammatory profiling with oxygenation metrics [30].

The cytokine-augmented models showed only modest gains in AUC. This is likely because the clinical anchors (SOFA score, immunosuppression, and hematologic malignancy) already captured most of the baseline risk, and because AUC is relatively insensitive to improvements concentrated in a small high-risk subgroup. The cohort of 41 patients with 15 ICU deaths provided about 4 events per variable and a non-trivial bootstrap optimism of 0.06–0.07 in apparent discrimination. The estimates are therefore unstable, and the findings should be regarded as exploratory and hypothesis-generating.

Several limitations should be noted. Antibiotics, vasoactive agents, and corticosteroids started before sampling could not be formally adjusted for in the analysis. Our cohort was recruited from a single tertiary ICU with a high proportion of immunocompromised patients, and the case mix may therefore limit the generalizability of our findings to other ICU settings.

Strengths of this study include prospective enrollment with clinical trial registration, standardized sampling on the day of ARF diagnosis, and the use of multiplex assays that reflect real-world ICU constraints.

## 5. Conclusions

In conclusion, this exploratory study identified monocyte- and macrophage-derived mediators (CXCL10, CCL2, IL-18, and IL-1Ra) as candidate prognostic biomarkers in heterogeneous ARF. These markers of early innate immune activation were elevated in non-survivors despite comparable clinical severity scores and oxygenation indices, with high-quartile risk enrichment most evident for IL-1Ra, CXCL10, and CCL2. These findings should be regarded as hypothesis-generating and require external validation in larger, independent cohorts.

## Figures and Tables

**Table 1:** Baseline characteristics and clinical outcomes according to ICU survival status.

	ICU Survival *N* = 26	ICU Non-Survival *N* = 15	*p*-Value
Age	65 [55, 71]	66 [61, 71]	0.800
Sex (Male, *n* (%))	16 (62%)	13 (87%)	0.200
SAPS-3, median (IQR)	60 [48, 75]	68 [62, 77]	0.078
SOFA score, median (IQR)	10.0 [7.0, 11.0]	11.0 [7.0, 16.0]	0.300
Solid cancer, *n* (%)	7 (27%)	4 (27%)	>0.900
Immunosuppression, *n* (%)	10 (38%)	12 (80%)	0.010
Hematologic malignancy, *n* (%)	8 (31%)	10 (67%)	0.026
Acute respiratory failure			0.600
ARDS, *n* (%)	3 (11.5)	1 (6.6)	
Aspiration, *n* (%)	3 (11.5)	0 (0)	
Pneumonia, *n* (%)	9 (34.6)	5 (33.4)	
Sepsis, *n* (%)	5 (19.2)	5 (33.4)	
Heart failure, *n* (%)	1 (3.9)	0 (0)	
Cardiogenic shock, *n* (%)	3 (11.5)	1 (6.6)	
Cardiac arrest, *n* (%)	1 (3.9)	0 (0)	
Others, *n* (%)	1 (3.9)	3 (20)	
Respiratory support			0.200
Mechanical ventilation, *n* (%)	17 (65%)	7 (47%)	
HFNC/NIV, *n* (%)	9 (35%)	8 (53%)	
PF ratio (mmHg), median (IQR)	178 [123, 226]	218 [114, 370]	0.400
PF ratio category, *n* (%)			0.200
≤100	3 (12%)	3 (20%)	
101–200	13 (50%)	4 (27%)	
201–300	6 (23%)	2 (13%)	
>300	4 (15%)	6 (40%)	
Intubated during ICU stay, *n* (%)	22 (85%)	15 (100%)	0.300
Extubation, *n* (%)	19 (86%)	1 (6.7%)	<0.001
Time to extubation, median (IQR)	3 [1, 4]	4 [4, 4]	0.900
Re-intubation, *n*	2	1	
Tracheostomy, *n*	4	6	
ICU length of stay (day), median (IQR)	7 [4, 12]	9 [3, 15]	0.600
Hospital length of stay (day), median (IQR)	25 [17, 39]	16 [6, 28]	0.032
In-hospital death, *n* (%)	4 (15%)	15 (100%)	<0.001

Values are presented as median [interquartile range] or number (percentage), as appropriate. *p*-values were calculated using the Mann–Whitney U test for continuous variables and the chi-squared or Fisher’s exact test for categorical variables. Abbreviations: ICU, intensive care unit; SAPS-3, Simplified Acute Physiology Score 3; SOFA, Sequential Organ Failure Assessment; HFNC, high-flow nasal cannula.

**Table 2:** Cytokine and biomarker levels at ICU admission according to ICU survival status.

Biomarker	ICU Survival (*N* = 26)	ICU Non-Survival (*N* = 15)	*p*-Value
IFN-γ	1 [0–3]	6 [1–20]	0.100
IL-1Ra	995 [628–2712]	7918 [1109–16,020]	0.067
IL-2	0.0 [0.0–1.6]	3.1 [0.0–4.1]	0.150
IL-4	0.00 [0.00–0.81]	0.69 [0.00–0.91]	0.400
IL-6	68 [32–877]	204 [23–3502]	0.500
IL-10	7 [5–20]	25 [6–233]	0.053
IL-13	0 [0–20]	38 [0–44]	0.067
IL-17A	0.0 [0.0–2.1]	0.1 [0.0–3.5]	0.300
IL-18	285 [140–814]	717 [416–2119]	0.009
IL-23	40 [0–84]	62 [0–210]	0.200
TNF-α	10 [6–23]	23 [13–144]	0.023
CCL2 (MCP-1)	178 [120–359]	768 [159–3872]	0.032
CCL3 (MIP-1α)	5 [0–22]	30 [6–85]	0.012
CCL4 (MIP-1β)	70 [0–138]	157 [64–541]	0.033
CXCL10 (IP-10)	143 [45–211]	353 [182–2125]	0.006
CCL5 (RANTES)	1747 [869–12,651]	2472 [1287–5306]	0.700
Angiopoietin-2	12,401 [5797–34,044]	17,549 [6484–35,260]	0.800
RAGE	4860 [3148–10,164]	10,805 [3445–27,221]	0.110
CA15-3 (MUC-1)	23 [13–47]	23 [18–52]	0.400

Units: pg/mL. Values are presented as median [interquartile range]. Cytokine and chemokine concentrations were measured in plasma at ICU admission. *p*-values were calculated using the Mann–Whitney U test. Abbreviations: IFN, interferon; IL, interleukin; TNF, tumor necrosis factor; CCL, CC chemokine ligand; CXCL, CXC chemokine ligand; MCP-1, monocyte chemoattractant protein-1; MIP, macrophage inflammatory protein; RAGE, receptor for advanced glycation end products.

**Table 3:** Univariable logistic regression for ICU mortality.

Variable	Univariable OR (95% CI)	*p*-Value
Clinical variables		
Age, per 1-year increase	1.00 (0.95–1.05)	0.860
Sex (Female vs. Male)	0.25 (0.03–1.15)	0.103
SAPS-3, per 1-point increase	1.03 (0.99–1.08)	0.153
SOFA, per 1-point increase	1.10 (0.94–1.32)	0.248
Immunosuppression	6.40 (1.58–33.56)	0.015
Hematologic malignancy	4.50 (1.21–18.86)	0.030
Biomarkers (per doubling, log2)		
CXCL10 (IP-10), per doubling (log2)	1.54 (1.12–2.27)	0.015
IL-18, per doubling (log2)	1.61 (1.08–2.57)	0.027
CCL2 (MCP-1), per doubling (log2)	1.53 (1.08–2.25)	0.022
IL-1Ra, per doubling (log2)	1.43 (1.03–2.05)	0.040

Values are odds ratios (OR) with 95% confidence intervals (CI) derived from univariable logistic regression models with ICU mortality as the dependent variable. Values below the LLOQ were imputed as LLOQ/2 after applying dilution factors; analytes with >30% of measurements below LLOQ were excluded from primary regression models. Cytokine concentrations were log2-transformed and expressed as OR per doubling of biomarker level. Abbreviations: ICU, intensive care unit; SAPS-3, Simplified Acute Physiology Score 3; SOFA, Sequential Organ Failure Assessment; OR, odds ratio; CI, confidence interval.

**Table 4:** Discrimination and calibration of SOFA-based clinical and cytokine-augmented models for ICU mortality.

Model	AUC (95% CI)	HL χ^2^ (df)	*p*-Value
SOFA only	0.60 (0.41–0.79)	6.66 (5)	0.247
Clinical core	0.74 (0.58–0.90)	5.74 (6)	0.453
Core + CXCL10 (IP-10, log2)	0.79 (0.63–0.95)	2.88 (6)	0.824
Core + IL-18 (log2)	0.76 (0.59–0.92)	8.15 (6)	0.228
Core + CCL2 (MCP-1, log2)	0.80 (0.66–0.93)	5.22 (6)	0.515
Core + IL-1Ra (log2)	0.78 (0.64–0.93)	3.19 (6)	0.785

All models used ICU mortality as the dependent variable and were fitted using logistic regression. The Clinical Core model included SOFA score, immunosuppression, and hematologic malignancy as covariates. Cytokine-augmented models additionally included one log2-transformed cytokine per doubling in concentration. Abbreviations: ICU, intensive care unit; SOFA, Sequential Organ Failure Assessment; AUC, area under the receiver operating characteristic curve; CI, confidence interval; HL, Hosmer-Lemeshow.

## Data Availability

The data presented in this study are available on request from the corresponding author due to privacy.

## Supplementary Materials

The following supporting information can be downloaded at https://www.mdpi.com/article/10.3390/diagnostics16121814/s1: Table S1: Baseline characteristics and outcomes by day 0 respiratory support modality; Table S2: Subgroup univariable logistic regression for ICU mortality by infection and by immunocompromised status; Table S3: Univariable logistic regression for ICU mortality across the full biomarker panel; Table S4: Multiple testing correction for univariable associations between cytokines and ICU mortality; Table S5: Bootstrap optimism-corrected discrimination of core and cytokine-augmented models for ICU mortality; Table S6: Sensitivity analysis. Univariable logistic regression for ICU mortality restricted to patients without hematologic malignancy; Table S7: Continuous variable Cox proportional hazards regression of time to ICU death; Figure S1: Plasma cytokine levels stratified by PaO_2_/FiO_2_ ratio category at ICU admission.

## Abbreviations

The following abbreviations are used in this manuscript:

ARDS	Acute Respiratory Distress Syndrome
ARF	Acute Respiratory Failure
AUC	Area Under the (Receiver Operating Characteristic) Curve
BH	Benjamini–Hochberg
CA15-3	Cancer Antigen 15-3
CI	Confidence Interval
COVID-19	Coronavirus Disease 2019
FiO_2_	Fraction of Inspired Oxygen
HFNC	High-Flow Nasal Cannula
ICU	Intensive Care Unit
IFN	Interferon
IL	Interleukin
IL-1Ra	Interleukin-1 Receptor Antagonist
IP-10	Interferon-Inducible Protein 10
IQR	Interquartile Range
IRB	Institutional Review Board
LLOQ	Lower Limit of Quantification
MCP-1	Monocyte Chemoattractant Protein-1
MIP	Macrophage Inflammatory Protein
OR	Odds Ratio
PaO_2_	Partial Pressure of Arterial Oxygen
P/F	PaO_2_/FiO_2_ ratio
RAGE	Receptor for Advanced Glycation End Products
SAPS-3	Simplified Acute Physiology Score 3
SOFA	Sequential Organ Failure Assessment
TNF	Tumor Necrosis Factor
