# Supplementary material for: Admission Cytokine Profiling for ICU Mortality Prediction in Heterogeneous Acute Respiratory Failure: An Exploratory Cytokine Profiling Study
Source: Diagnostics (Basel). 2026 Jun 12;16(12):1814. doi: 10.3390/diagnostics16121814 (PMC13298018; doi:10.3390/diagnostics16121814)
Supplement: Supplementary file 1 [file diagnostics-16-01814-s001.zip › diagnostics-4354059-supplementary.pdf]

Supplementary Materials

Admission Cytokine Profiling for ICU Mortality Prediction in Heterogeneous Acute Respiratory Failure

Table S1. Baseline characteristics and outcomes by day 0 respiratory support modality

Variable	MV (n=24)	HFNC/NIV (n=17)	p value
Age, years	65 (59–75)	66 (52–69)	0.412
Male sex	17 (71%)	12 (71%)	1.000
SAPS3	64 (57–80)	62 (51–69)	0.375
SOFA	10 (8–12)	10 (6–11)	0.196
PaO_2/FiO_2 ratio	183 (125–292)	166 (123–268)	0.947
Immunosuppression	13 (54%)	9 (53%)	1.000
Hematologic malignancy	10 (42%)	8 (47%)	0.760
Solid cancer	6 (25%)	5 (29%)	1.000
Infectious ARF etiology	13 (54%)	11 (65%)	0.539
ICU mortality	7 (29%)	8 (47%)	0.328
Hospital mortality	11 (46%)	8 (47%)	1.000
ICU length of stay, days	8 (5–14)	6 (3–13)	0.771

Continuous variables are presented as median (interquartile range) and compared by the Mann–Whitney U test. Categorical variables are presented as n (%) and compared by Fisher's exact test (2x2) or chi-squared test (otherwise). PaO_2/FiO_2 ratio was computed from PaO_2 measured at day 0 and the corresponding ventilator FiO_2 (mechanically ventilated patients) or HFNC/NIV FiO_2. MV, invasive mechanical ventilation; HFNC, high-flow nasal cannula; NIV, non-invasive ventilation; SAPS3, Simplified Acute Physiology Score 3; SOFA, Sequential Organ Failure Assessment.

Table S2. Subgroup univariable logistic regression for ICU mortality by infection and by immunocompromised status

Cytokine	Infectious (n=24, 10 deaths) OR (95% CI)	p	Non-infectious (n=17, 5 deaths) OR (95% CI)	p	Cytokine × infection p	Immunocompromised (n=22, 12 deaths) OR (95% CI)	p	Non-immunocompromised (n=19, 3 deaths) OR (95% CI)	p	Cytokine × immunocompromise p
CXCL10 (IP-10)	1.39 (0.94–2.08)	0.102	1.96 (0.98–3.93)	0.058	0.427	1.49 (0.84–2.65)	0.171	1.27 (0.76–2.12)	0.357	0.685
CCL2 (MCP-1)	1.47 (0.97–2.21)	0.068	1.67 (0.76–3.70)	0.204	0.806	1.16 (0.74–1.81)	0.519	1.94 (0.79–4.74)	0.147	0.315
IL-18	1.39 (0.84–2.31)	0.200	2.00 (0.93–4.28)	0.076	0.444	1.26 (0.69–2.29)	0.447	1.53 (0.72–3.26)	0.267	0.702
IL-1RA	1.38 (0.90–2.12)	0.144	1.47 (0.81–2.64)	0.202	0.852	1.11 (0.74–1.69)	0.610	2.04 (0.96–4.32)	0.063	0.216
TNF-α	1.51 (0.97–2.36)	0.070	2.07 (0.69–6.22)	0.193	0.590	1.24 (0.73–2.09)	0.428	1.71 (0.75–3.90)	0.201	0.546
IL-6	1.05 (0.83–1.33)	0.662	1.07 (0.77–1.48)	0.692	0.939	0.97 (0.78–1.21)	0.806	1.12 (0.69–1.82)	0.659	0.621

Univariable logistic regression for in-ICU mortality stratified by subgroup. OR with 95% CI are per log2 doubling of plasma concentration. Infectious ARF is defined as pneumonia or sepsis. Interaction p values are from SOFA adjusted logistic regression with a cytokine by subgroup interaction term. Abbreviations: ARF, acute respiratory failure; ICU, intensive care unit; OR, odds ratio; CI, confidence interval; SOFA, Sequential Organ Failure Assessment; CXCL10, C-X-C motif chemokine ligand 10 (IP-10); CCL2, C-C motif chemokine ligand 2 (MCP-1); IL, interleukin; IL-1Ra, interleukin-1 receptor antagonist; TNF, tumor necrosis factor.

Table S3. Univariable logistic regression for ICU mortality across the full biomarker panel

Biomarker	OR (95% CI)	p value	AUC	% below LLOQ	Retained
CXCL10 (IP-10)	1.54 (1.12–2.27)	0.015	0.76	0.0	Yes
IL-18	1.61 (1.08–2.57)	0.027	0.75	0.0	Yes
IL-10	1.41 (1.06–2.00)	0.028	0.71	41.5	No
CCL2 (MCP-1)	1.53 (1.08–2.25)	0.022	0.70	0.0	Yes
CCL3 (MIP-1α)	1.54 (1.08–2.36)	0.028	0.70	51.2	No
IL-1RA	1.43 (1.03–2.05)	0.040	0.67	0.0	Yes
CCL4 (MIP-1β)	2.03 (1.09–4.17)	0.033	0.66	73.2	No
IFN-γ	1.26 (1.01–1.65)	0.056	0.65	41.5	No
sRAGE	1.51 (0.95–2.52)	0.091	0.65	0.0	Yes
TNF-α	1.66 (1.07–2.80)	0.034	0.65	68.3	No
CA15-3 (MUC1)	1.30 (0.84–2.12)	0.252	0.58	7.3	Yes
IL-6	1.09 (0.91–1.31)	0.369	0.57	14.6	Yes
IL-23	Not estimable	0.997	0.57	95.1	No
IL-13	3.63 (0.52–61.55)	0.229	0.55	92.7	No
IL-2	1.29 (0.41–3.94)	0.641	0.54	85.4	No
Angiopoietin-2	1.01 (0.63–1.63)	0.976	0.52	0.0	Yes
IL-4	Not estimable	0.991	0.52	97.6	No
IL-17A	0.80 (0.10–2.02)	0.686	0.49	95.1	No
CCL5 (RANTES)	0.96 (0.68–1.34)	0.823	0.46	12.2	Yes

Univariable logistic regression for in-ICU mortality in 41 patients with 15 deaths. OR with 95% CI are per log2 doubling of plasma concentration. AUC is from the biomarker alone. The 'Retained' column indicates whether the analyte was included in primary regression analyses based on the >30% effective lower-limit-of-quantification (LLOQ) exclusion rule. 'Not estimable' indicates that the proportion of values below LLOQ was so high that the regression estimate was

singular. Abbreviations: ICU, intensive care unit; OR, odds ratio; CI, confidence interval; AUC, area under the receiver operating characteristic curve; LLOQ, lower limit of quantification; IL, interleukin; IL-1Ra, interleukin-1 receptor antagonist; IFN, interferon; TNF, tumor necrosis factor; CCL, C-C motif chemokine ligand; CXCL, C-X-C motif chemokine ligand; IP-10, interferon-inducible protein-10; MCP-1, monocyte chemoattractant protein-1; MIP, macrophage inflammatory protein; sRAGE, soluble receptor for advanced glycation end-products; CA15-3, cancer antigen 15-3; MUC1, mucin 1; RANTES, regulated upon activation, normal T-cell expressed and secreted.

Table S4. Multiple testing correction for univariable associations between cytokines and ICU mortality

Cytokine	Raw p	Benjamini–Hochberg q	Bonferroni-adjusted p
CXCL10 (IP-10)	0.015	0.082	0.131
CCL2 (MCP-1)	0.022	0.082	0.194
IL-18	0.027	0.082	0.245
IL-1RA	0.040	0.090	0.360
sRAGE	0.091	0.164	0.822
CA15-3 (MUC1)	0.252	0.378	>0.999
IL-6	0.369	0.475	>0.999
CCL5 (RANTES)	0.823	0.926	>0.999
Angiopoietin-2	0.976	0.976	>0.999

Multiple testing correction across the 9 cytokines retained after the greater than 30 percent lower limit of quantification filter. q value, Benjamini and Hochberg adjusted false discovery rate. Bonferroni-adjusted p values are also shown. Cytokines are sorted by raw p value. Abbreviations: ICU, intensive care unit; BH, Benjamini–Hochberg; CXCL10, C-X-C motif chemokine ligand 10 (IP-10); CCL2, C-C motif chemokine ligand 2 (MCP-1); IP-10, interferon-inducible protein-10; MCP-1, monocyte chemoattractant protein-1; IL, interleukin; IL-1Ra, interleukin-1 receptor antagonist; sRAGE, soluble receptor for advanced glycation end-products; CA15-3, cancer antigen 15-3; MUC1, mucin 1; RANTES, regulated upon activation, normal T-cell expressed and secreted.

Table S5. Bootstrap optimism-corrected discrimination of core and cytokine-augmented models for ICU mortality

Model	n	Apparent AUC	Optimism	Optimism-corrected AUC	95% CI
Core (SOFA+immunocomp+heme)	41	0.747	0.057	0.676	0.52–0.85
Core + CXCL10	41	0.779	0.066	0.706	0.57–0.85
Core + CCL2	41	0.790	0.069	0.713	0.56–0.85
Core + IL-18	41	0.762	0.070	0.668	0.51–0.83
Core + IL-1Ra	41	0.787	0.070	0.704	0.55–0.84

Internal validation by 1,000 iteration bootstrap with Harrell's optimism correction. Core model includes SOFA, immunosuppression, and hematologic malignancy, with one cytokine added separately on the log2 scale. The 95% CI is the percentile interval of the optimism-corrected AUC. Abbreviations: ICU, intensive care unit; SOFA, Sequential Organ Failure Assessment; AUC, area under the receiver operating characteristic curve; CI, confidence interval; CXCL10, C-X-C motif chemokine ligand 10 (IP-10); CCL2, C-C motif chemokine ligand 2 (MCP-1); IL, interleukin; IL-1Ra, interleukin-1 receptor antagonist.

Table S6. Sensitivity analysis. Univariable logistic regression for ICU mortality restricted to patients without hematologic malignancy

Cytokine	OR (95% CI)	p value	Benjamini–Hochberg q
CCL2 (MCP-1)	1.72 (0.98–3.37)	0.071	0.315
CXCL10 (IP-10)	1.71 (0.99–3.50)	0.077	0.315
IL-18	1.95 (0.91–4.90)	0.105	0.315
CA15-3 (MUC1)	1.49 (0.79–3.29)	0.254	0.471
IL-1RA	1.38 (0.78–2.50)	0.262	0.471
sRAGE	1.29 (0.63–2.71)	0.470	0.677
IL-6	1.09 (0.82–1.46)	0.526	0.677
CCL5 (RANTES)	1.10 (0.65–1.91)	0.712	0.744
Angiopoietin-2	1.14 (0.54–2.71)	0.744	0.744

Sensitivity analysis restricted to 23 patients without hematologic malignancy (5 in-ICU deaths). Univariable logistic regression per log2 doubling of plasma concentration. Analysis restricted to the 9 cytokines retained after the greater than 30 percent lower limit of quantification filter. q value, Benjamini and Hochberg adjusted false discovery rate. Abbreviations: ICU, intensive care unit; OR, odds ratio; CI, confidence interval; BH, Benjamini–Hochberg; CXCL10, C-X-C motif chemokine ligand 10 (IP-10); CCL2, C-C motif chemokine ligand 2 (MCP-1); IP-10, interferon-inducible protein-10; MCP-1, monocyte chemoattractant protein-1; IL, interleukin; IL-1Ra, interleukin-1 receptor antagonist; sRAGE, soluble receptor for advanced glycation end-products; CA15-3, cancer antigen 15-3; MUC1, mucin 1; RANTES, regulated upon activation, normal T-cell expressed and secreted.

Table S7. Continuous variable Cox proportional hazards regression of time to ICU death

Cytokine	HR (95% CI)	p value	Benjamini–Hochberg q
IL-1RA	1.43 (1.09–1.87)	0.009	0.066
CCL2 (MCP-1)	1.41 (1.07–1.87)	0.015	0.066
sRAGE	1.72 (1.08–2.73)	0.022	0.066
CXCL10 (IP-10)	1.24 (1.00–1.55)	0.052	0.111
IL-6	1.15 (0.99–1.32)	0.061	0.111
IL-18	1.21 (0.94–1.57)	0.140	0.209
Angiopoietin-2	1.31 (0.88–1.94)	0.179	0.231
CCL5 (RANTES)	0.94 (0.71–1.24)	0.662	0.744
CA15-3 (MUC1)	1.06 (0.74–1.52)	0.757	0.757

Univariable Cox proportional hazards regression of time from ICU admission to in-ICU death, censored at ICU discharge. HR with 95% CI are per log2 doubling of plasma concentration. Analysis restricted to the 9 cytokines retained after the greater than 30 percent lower limit of quantification filter. q value, Benjamini and Hochberg adjusted false discovery rate. Abbreviations: ICU, intensive care unit; HR, hazard ratio; CI, confidence interval; CXCL10, C-X-C motif chemokine ligand 10 (IP-10); CCL2, C-C motif chemokine ligand 2 (MCP-1); IP-10, interferon-inducible protein-10; MCP-1, monocyte chemoattractant protein-1; IL, interleukin; IL-1Ra, interleukin-1 receptor antagonist; sRAGE, soluble receptor for advanced glycation end-products; CA15-3, cancer antigen 15-3; MUC1, mucin 1; RANTES, regulated upon activation, normal T-cell expressed and secreted.

Supplementary figure

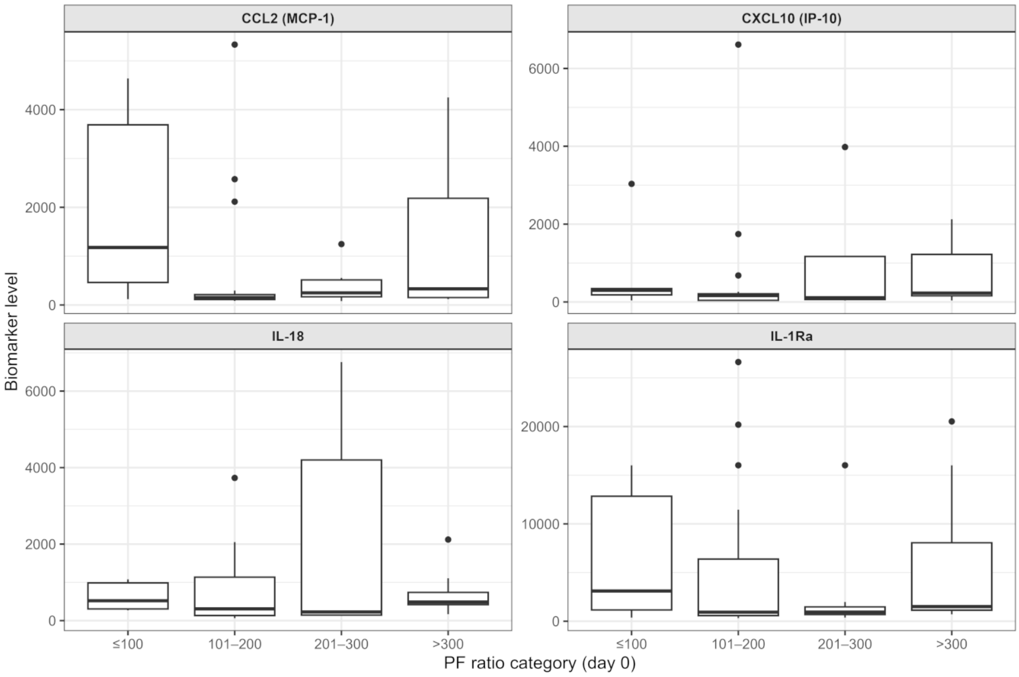

Figure S1. Plasma cytokine levels stratified by PaO_2/FiO_2 ratio category at ICU admission.

Box plots show plasma concentrations of CCL2 (MCP-1), CXCL10 (IP-10), IL-18, and IL-1Ra according to baseline PaO_2/FiO_2 ratio categories. Boxes represent interquartile ranges with median lines, whiskers extend to 1.5 times the interquartile range, and individual values are overlaid. Wide within category dispersion and the absence of a consistent graded trend suggest that systemic inflammatory profiles and oxygenation severity capture distinct dimensions of disease heterogeneity. The figure image is provided as a separate file (Supplementary Figure S1). Abbreviations: ICU, intensive care unit; PaO_2/FiO_2, ratio of arterial partial pressure of oxygen to fraction of inspired oxygen; CCL2, C-C motif chemokine ligand 2 (MCP-1); CXCL10, C-X-C motif chemokine ligand 10 (IP-10); IL-18, interleukin-18; IL-1Ra, interleukin-1 receptor antagonist.
